# War is not a pandemic: The collapse of scientific continuity under constant attack

**DOI:** 10.1038/s44319-026-00835-z

**Published:** 2026-06-15

**Authors:** Shai Berlin, Mykhailo O Kompanets

**Affiliations:** 1 Department of Neuroscience, Rappaport Faculty of Medicine, Technion – Israel Institute of Technology, Haifa, Israel; 2 L.M. Litvinenko Institute of Physico-Organic Chemistry and Coal Chemistry, National Academy of Sciences of Ukraine, Kyiv, Ukraine

**Keywords:** History & Philosophy of Science, Methods & Resources, Science Policy & Publishing

## Abstract

The ongoing wars in Ukraine and the Middle East have greatly damaged science but also provided valuable lessons on increasing resilience during wartime.

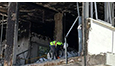

## Shai Berlin

As the week draws to a close, I review ongoing work, prioritize tasks that require immediate attention—even over the weekend—versus those that can wait until next week. The list is familiar: grant deadlines, manuscript revisions, committee work, teaching and finalizing travel arrangements for upcoming conferences. As I exit my office, a PhD student approaches to confirm my availability next week for a time-sensitive experiment. I answer positively, as I welcome any opportunity to step away from the computer. Moments later, my postdoctoral fellow updates me that our transgenic mouse line has produced sufficient litters, stereotaxic injections have been completed, and cultures suitable for electrophysiological recordings are prepared. These data are critical for a patent filing and as preliminary results for a grant. Overall, things couldn’t be better—what a great way to start the weekend.

Two days later, on Saturday, February 28th, this uplifting mood evaporates. Air-raid sirens sound, and we move to the shelter. Within that confined space, conversations with my wife quickly shift to logistics. Almost reflexively, we revert to familiar COVID-like terminology—“capsules”: I organize my students into small rotating groups to maintain essential laboratory operations, while my wife and I coordinate rotating work shifts. We also anticipate that the children will attend school in small groups on alternating days, if schools remain open at all. In parallel, we expect a return to remote schooling for the children and online teaching for me via video conferencing platforms. I immediately begin to notify conference organizers of my withdrawal, request indefinite extensions from journal editors, and inform my students—reluctantly—that all experiments must be suspended indefinitely. Missed deadlines, delayed patents and uncertainty become inevitable.

## Mykhailo Kompanets

**Figure 1:**
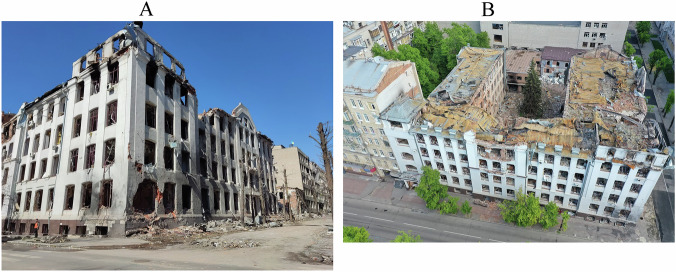
Destruction in the Ukraine. (**A**) The building of the Institute of Economics of the Karazin Kharkiv National University damaged by a Russian missile on March 2, 2022. Aerial photo is taken from our drone on May 13, 2022. (**B**) Institute of State Management of the Karazin Kharkiv National University badly damaged by a Russian missile on March 18, 2022. Credits: With permission by the Maidan Monitoring Information Centre. https://maidan.org.ua/.

The date February 28th carries an unsettling resonance: on February 24, 2022, Russia began its full-scale invasion of Ukraine. What had been a simmering regional confrontation for 12 years, transformed into a devastating war, leading to systemic breakdown of the entire national research ecosystem. In the early days of the war, we assumed that the shooting would not go on for much longer and pursued our research as best as we could. However, gunfire was followed by artillery shelling and indiscriminate bombing of civilian areas. With no electricity, heating or running water, work in the laboratory became increasingly difficult (Fig. 1) and ultimately came to a complete halt, followed by widespread evacuations from the cities. I therefore closed the lab, dismissed the few remaining students, and went home. Official guidance from Ukrainian universities on how to proceed under these circumstances was limited and our decisions were largely shaped through fragmented exchanges with colleagues, supplemented by our own personal experiences with major disruptions, most notably the COVID-19 pandemic.

With no electricity, heating or running water, work in the laboratory became increasingly difficult and ultimately came to a complete halt…

## The preceding pandemic

It is neither surprising, nor coincidental, that we both relate our current working conditions to those we experienced during COVID-19; in fact, our scientific collaboration began just as the pandemic unfolded. That period had a profound impact on our teamwork, largely preventing any face-to-face meetings. Nevertheless, we kept working together and exchanging ideas, primarily through online platforms. Having navigated the disruptions brought about by COVID for 2 years, we initially felt confident in our ability to adapt to wartime conditions; however, we soon realized that this was a completely different challenge.

The disruption of research during the COVID-19 pandemic was nothing short of momentous, leading to sharp declines in research activities, with 70–80% of wet-lab work halted and global scientific output falling by 20–30% in 2020 (Myers et al, 2020). Beyond the immediate closure of laboratories, the pandemic caused widespread delays in ongoing experiments, loss of valuable biological samples, and interruptions of long-term studies that could not simply be paused and resumed. Access to core facilities and shared equipment was severely restricted, and supply chain disruptions led to shortages of essential reagents and materials. Collaborations, such as our own, also suffered as travel restrictions and the impossibility of in-person meetings limited the exchange of ideas and slowed the establishment of new partnerships. These factors created a sustained and uneven slowdown in research that extended well beyond the initial lockdowns. However, the similarities between then and now end there.

The speed and magnitude of the collapse in the wake of war quickly made it clear that these are not disruptions we had previously “learned” to manage. In fact, during the pandemic, most experimental systems were preserved and recovery from the lockdowns, albeit slow, eventually brought labs back to full work. Importantly, the demographic distribution of COVID-19 mortality meant that the large majority of scientists were comparatively spared, enabling many of them to return to work immediately (O’Driscoll et al, 2021). COVID-19 had temporarily paused research, but it had not disrupted it. Wars introduce a fundamentally different situation—one that actively erodes scientific research, personnel and infrastructure. It combines the constraints such as during the pandemic with far more severe challenges: physical destruction, loss of infrastructure, loss of human capital and pervasive insecurity.

The speed and magnitude of the collapse in the wake of war quickly made it clear that these are not disruptions we had previously “learned” to manage.

Wars introduce a fundamentally different situation—one that actively erodes scientific research, personnel and infrastructure.

**Figure 2:**
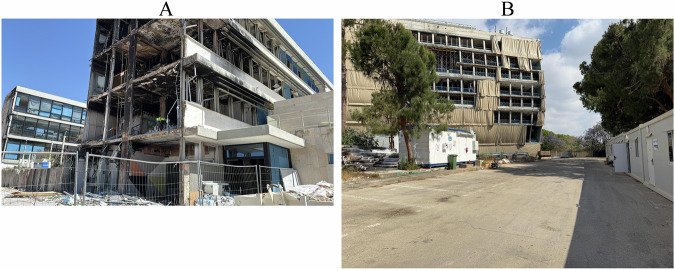
Iranian missile strike on the Weizmann Institute of Science, Israel. (**A**) The main building of the Weizmann Institute in Rechovot, Israel, after it was severely damaged by Iranian missiles on June 15, 2025. Photo credit: Michal Rivlin, Weizmann Institute. (**B**) The same building undergoing reconstruction in May 2026. Photo credit: Michal Rivlin, Weizmann Institute.

In Israel, Iranian missile strikes have hit and damaged research institutions, including the Weizmann Institute of Science, with losses estimated in the hundreds of millions of dollars (Tercatin, 2025) (Fig. 2, A). Beyond the destruction of equipment and buildings, the more profound loss lies in years of accumulated biological and other research materials, as well as complex experimental systems—many of which are irreplaceable. Some Israeli researchers have stated that continuing their previous lines of inquiry would effectively require them to start from scratch, leading them to consider abandoning them altogether. Ukraine, has suffered a similar but far more extensive disruption with more than 1200 scientific and educational institutions damaged or destroyed. In such settings, recovery extends far beyond rebuilding physical infrastructure; it necessitates the reconstruction of entire intellectual ecosystems, including researchers, students and other personnel, and requires substantial resources and long timescales.

Even when infrastructure remains intact, the conditions necessary for scientific work are profoundly compromised. Laboratory operations, sometimes organized into shifts or “capsules” may resemble pandemic-era adaptations—but with a critical difference: unpredictability. Air-raid sirens, evacuations and other threats disrupt experiments without warning, often multiple times a day. These interruptions do not merely introduce variability and failure; they further impose a psychological burden. Researchers report growing exhaustion, disengagement and declining motivation. Experiments are abandoned midway. Workdays fragment. Attention is divided between research and the safety of family members. For scientists who are parents, the burden is especially acute. Unlike the pandemic—which posed relatively low direct risk to children and allowed for more structured childcare decisions—war places children in immediate danger. Schooling becomes unstable, and informal support networks such as grandparents or extended family are often not available. The consequence is not simply reduced productivity, but a diminished ability to attend work consistently or sustain meaningful research activity.

Crucially, wartime disruption directly affects scientists themselves. In Israel, a substantial proportion of the academic workforce—including students and early-career researchers—has been mobilized as reservists in the army. Estimates suggest that 18–30% of university students have been called to service (Fiske, 2023). Some have not returned. Ukraine faced even greater losses through displacement and attrition, with around 10–20% of researchers forced to leave their positions and ~18% of the scientific workforce lost during the war (De Rassenfosse et al, 2023). These conflicts therefore erode not only experimental systems, but also human capital.

The distinction is therefore not only quantitative, but qualitative. Pandemic-related disruptions constrained productivity. War disrupts the very conditions that make science possible.

## Preparing for disruption

Lessons from recent armed conflicts, particularly in Ukraine, suggest that strengthening and sustaining scientific research during wartime requires a fundamental restructuring of research systems. Distributed and decentralized infrastructure—where critical materials and data are duplicated across institutions—help to enhance resilience. International collaborations and pre-arranged “host laboratories” could also help to maintain continuity when local facilities are destroyed or become inoperative. Nonetheless, such strategies still have limitations. Duplicating laboratory infrastructure is costly, space-intensive and logistically complex. Maintaining parallel systems requires additional personnel and sustained funding. Safeguarding irreplaceable materials—through cryopreservation, biobanking and off-site storage by centralized dedicated facilities—may therefore be more effective. Moreover, relocation is often impractical. Airspace closures or destruction of roads and railways limit mobility. In addition, societal cohesion and national resilience, such as in Israel, mean that many researchers prefer to stay at home despite ongoing conflict.

Instead, there should be more emphasis on protecting and retaining personnel. Wartime attrition is not temporary and it risks permanently weakening the scientific workforce. As highlighted by UNESCO’s action plan for Ukraine, sustaining science during war requires investment in people: salary continuity, emergency support for trainees, reduced administrative burdens and structured re-entry pathways for those returning from reserve duty or prolonged displacement.

Wartime attrition is not temporary and it risks permanently weakening the scientific workforce.

Finally, one of the most critical—yet frequently overlooked—challenges is funding. During conflict, national priorities shift toward security and the military, leaving research systems with limited flexibility. Funding structures must therefore be redesigned to recognize war as a distinct category of disruption. Conventional extensions are insufficient. What may be needed are rapid-response continuity grants, automatic no-cost extensions, emergency salary support, dedicated resources for specimen preservation and mechanisms to compensate for lost experiments.

## War is not a pandemic

Importantly, lessons from the COVID-19 pandemic should not be adopted uncritically when designing responses to wartime disruption. While the pandemic required rapid, reactive reallocation of funding and research efforts toward solving a common, time-limited global problem, it did not fundamentally compromise the infrastructure of science itself or the safety of the scientific workforce. War, on the other hand, represents a fundamentally different and more persistent challenge. It actively degrades infrastructure, disperses or depletes the scientific workforce, and disrupts access to essential tools and materials.

As such, wartime science policy should not prioritize short-term redirection of research efforts toward specific topics, but rather long-term preparedness and resilience. If the pandemic taught us how to respond to sudden, global disruptions, ongoing armed conflicts such as those in Ukraine and Israel highlight a different imperative: how to prepare research systems to withstand sustained and widespread interruption. The challenge is, therefore, not simply to restart laboratories after conflict, but to design systems capable of absorbing disruption without losing people, materials or institutional memory.

The challenge is […]to design systems capable of absorbing disruption without losing people, materials or institutional memory.

And yet, science persists in Israel and Ukraine (Fig. 2, B). As during the pandemic, the current wars highlight the resilience of scientists and the research systems. In Israel, most laboratories have resumed their activities. In Ukraine, research continues wherever possible, often in fragmented or relocated forms despite ongoing bombardments. Scientific progress does not stop—it changes and recalibrates but ultimately keeps moving forward. With timely and targeted support from local institutions and the international community, the continuity of science can be maintained even under the most adverse circumstances. Still, some things remain unchanged—we have still not managed to meet face-to-face, not even a single time, since our first online encounter during the pandemic, some five years ago.

## Supplementary information

Peer Review File
