# Supplementary material for: War is not a pandemic: The collapse of scientific continuity under constant attack
Source: EMBO Rep. 2026 Jun 15;27(13):3513–6. doi: 10.1038/s44319-026-00835-z (PMC13354767; doi:10.1038/s44319-026-00835-z)
Supplement: Supplementary file 1 — Peer Review File [file 44319_2026_835_MOESM1_ESM.pdf]

EMBO reports

War is not a pandemic: The collapse of scientific continuity under constant attack

Shai Berlin and Mykhailo Kompanets

Corresponding author(s): Shai Berlin (shai.berlin@technion.ac.il)

Review Timeline:

Submission Date:	26th May 26
Accepted:	5th Jun 26

Editor: Holger Breithaupt

Transaction Report: This article was editorially reviewed.
